# Maternal Financial Autonomy as Predictor of Children's Meal Frequency: Evidence from Jimma Zone, Oromia, Ethiopia

**DOI:** 10.4314/ejhs.v35i1.3

**Published:** 2025-01

**Authors:** Jemal Abafita

**Affiliations:** 1 Department of Economics, College of Business and Economics, Jimma University, Jimma, Ethiopia

**Keywords:** Maternal financial autonomy, Principal Component Analysis, Women's economic freedom, Child nutritional outcomes, Children's meal frequency, Jimma, Ethiopia

## Abstract

**Background:**

Optimal meal frequency is a key factor in determining nutritional outcomes for infants and young children (6 to 24 months). While previous studies have emphasized household socio-demographics and healthcare utilization as major influences on child feeding practices, fewer have focused on intrinsic maternal factors such as financial autonomy. This study explored the relationship between maternal financial autonomy and child meal frequency.

**Methods:**

A cross-sectional, community-based study was conducted in Jimma Zone, Southwest Ethiopia. A multi-stage stratified sample of 572 participants was selected from three Woredas (Mana, Gomma, and Limukossa), stratified by semiurban and rural areas. Data were collected through face-to-face interviews with women caregivers using a structured questionnaire. Maternal financial autonomy was assessed across four domains adapted from the Demographic and Health Survey (DHS) tool, while children's meal frequency was evaluated based on WHO Infant and Young Children Feeding (IYCF) recommendations. Multivariable logistic regression was used for analysis.

**Results:**

Among the mothers, 61.3% had the freedom to visit marketplaces, and 80.1% had the autonomy to purchase food. However, only 43.6% had autonomy over major household purchases, and 45.3% were able to work outside the home. Overall, 51.6% of the 548 mothers interviewed scored below half in the four autonomy domains. Over half (52.9%) of the children had suboptimal meal frequency. After adjusting for factors such as setting, family size, household head's sex, child sex and age, marital status, land ownership, wealth index, dependency ratio, and maternal education, maternal financial autonomy was strongly associated with improved child meal frequency (OR 5.90, 95% CI: 3.97 to 8.78).

**Conclusion:**

Maternal financial autonomy was strongly linked to child meal frequency in the study area. Interventions should focus on enhancing women's control over resources in addition to addressing health and food security issues.

## Introduction

Child undernutrition remains a major global challenge, with stunting affecting over 22% of children worldwide and wasting impacting around 6.7% (1). In Africa, malnutrition rates are disproportionately high, with over 30% of children under five stunted and approximately 8% wasted, according to a 2023 UNICEF report (2-3). Contributing factors include food insecurity, limited healthcare access, poor feeding practices, and socioeconomic challenges (4).

In Ethiopia, child malnutrition is a significant public health concern. The 2019 Ethiopian Demographic and Health Survey reveals that about 37% of children under five are stunted, reflecting widespread food insecurity and famine conditions (5). This issue is driven by inadequate feeding practices and illnesses related to unfavorable environmental and socioeconomic factors (4-6). Ensuring a minimum acceptable diet, which includes dietary diversity and adequate feeding frequency, is crucial to preventing the severe consequences of malnutrition (6). However, in Ethiopia, only 55% of children meet the recommended meal frequency (5, 7).

Meal frequency, which refers to the number of times a child receives solid, semi-solid, or soft foods daily, is a key indicator of nutritional intake and health. WHO guidelines recommend that infants aged 6-8 months receive solid foods at least twice daily. Breastfed children aged 9-23 months should have a frequency of at least three times daily, while non-breastfed children in the same age group should receive these foods at least four times daily (8, 9).

Several household-level factors influencing meal frequency, such as low socioeconomic status and food insecurity, have been documented (7, 10-11). However, maternal factors, particularly financial autonomy, have received less attention. Maternal financial autonomy significantly impacts child feeding practices by enabling mothers to make independent purchasing decisions, improving access to nutrient-rich foods, and enhancing overall diet quality (12-15). When women control household finances, they are more likely to allocate resources toward essential food items and healthcare services, resulting in better health and nutritional outcomes for their children. Financial control also reduces food insecurity, ensuring consistent access to adequate nutrition (14). Moreover, it empowers mothers to make informed feeding decisions and challenge cultural norms that may hinder optimal feeding practices (12-13). Reduced financial stress can further enhance maternal mental health, leading to more responsive and attentive feeding practices, ultimately supporting improved child nutrition (16).

In Ethiopia, maternal financial autonomy is often limited by socio-economic and cultural barriers. Women frequently face restricted access to income-generating opportunities, traditional gender roles that limit their financial decision-making, and limited control over household resources (17, 18). These factors may hinder a mother's ability to ensure her child receives the recommended minimum meal frequency. This study explores the relationship between maternal financial autonomy and children's meal frequency in the Jimma Zone of Ethiopia.

## Materials and Methods

**Study setting and design**: A cross-sectional, community-bed in Jimma Zone, Southwest Ethiopia. Jimma is part of the Oromia region and is known for its rich biodiversity, particularly in Arabica coffee cultivation. The zone has a population of 2,495,795, as per the 2007 national census, and covers an area of 15,569 square kilometers.

**Sample size and sampling procedure**: Sample size was calculated based on the proportion of Ethiopian children aged who met the minimum meal frequency (55%), with a design effect of 1.5 and a margin of error of 0.05. Using Epi Info Verstotal sample size required was 572 to achieve 80% power. The study excluded children with severe acute malnutrition, serious illnesses requiring hospitalization, or congenital/chronic conditions affecting feeding or growth. A multi-stage stratified sampling method was used, selecting three Woredas (Mana, Gomma, and Limukossa) from the nine predominantly cash-cropping districts. These Woredas were stratified by urban and rural areas, with one-third of villages (Gots) and kebeles selected as primary sampling units. Households with eligible children were randomly chosen using a registry from health extension workers. Proportional allocation was based on the 2007 Central Statistics Agency report, with the youngest eligible child selected from households with multiple children.

**Data collection and analysis**: Data were collected through face-to-face interviews with mothers or caregivers using a structured questionnaire. Ethical approval was granted by the Institutional Review Board of Jimma University, Ethiopia. Informed verbal and written consent was obtained from participants.

The wealth index was determined using Principal Component Analysis (PCA), with scores for 25 asset types converted into latent factors. The first factor was used to categorize households into wealth tertiles. Multivariable logistic regression analysis (SPSS Version 21) assessed the association between maternal financial autonomy and children's minimum meal frequency, adjusting for relevant covariates. Household food security was assessed using the Household Food Insecurity Access Scale (HFIAS) Version 3, developed by FAO and FANTA. Meal frequency was assessed using WHO IYCF feeding recommendations. Maternal financial autonomy was measured using four domains adapted from the DHS tool, evaluating women's autonomy in marketplace activities, purchasing food, acquiring major household items, anutside the home. Achieving at least half of these domains indicated autonomy.

## Results

As presented in Table 1, a total of 548 households were surveyed, with a response rate of 96%. The sample predominantly comprises rural households, with 75.2% (412 households) located in rural areas and 24.8% (136 households) in semi-urban areas. Religious distribution shows that 74.8% (410 households) are Muslim, 14.2% (78 households) are Orthodox Christians, and 10.9% (60 households) are Protestant Christians. The majority ethnic group is Oromo, comprising 76.5% (419 households), followed by Amhara (8.0%, 44 households), Silte (7.3%, 40 households), Dawro (6.6%, 36 households), and other ethnic groups (2.7%, 15 households).

**Table 1:** Socio-demographic characteristics of Households, Jimma Zone, Ethiopia, 2023

Variables		Total	Percent or Mean ± Sd
**Setting**	Rural	412	75.2
	Semi Urban	136	24.8
**Religion**	Muslim	410	74.8
	Orthodox Christians	78	14.2
	Protestant	60	10.9
**Household Ethnicity**	Oromo	419	76.5
	Amhara	44	8.0
	Silte	40	7.3
	Dawro	36	6.6
	Others	15	2.7
**Marital Status**	Married	502	91.6
	Divorced/ Widowed	46	8.4
**Sex Of The Household Head**	Male	518	94.5
	Female	30	5.5
**HHD educational status**	No formal education	120	21.9
	Primary/Secondary	382	69.7
	Collage/university	46	8.4
**Spouse educational status**	No formal education	168	30.7
	Primary/Secondary	352	64.2
	Collage/university	28	5.1
**Food insecurity**	Insecure	373	68.1
	secure	175	31.9
**Wealth Index**	Higher	134	24.5
	Lower	196	35.8
	Medium	218	39.8
**Land ownership**	Yes	286	52.2
	No	262	47.8
**Family Size** (Mean± Sd)			5.1±1.8
**Mean of Age Dependency ratio** (Mean± Sd)			0.5±0.2
**Mean of paternal-maternal age difference** (Mean± Sd)			7.6 ±6.0
**Mean dependency ratio** (Mean± Sd)			2.2 ±1.3
**Land size** (Mean± Sd)			0.43±22

In terms of marital status, 91.6% (502 households) are married, while 8.4% (46 households) are divorced or widowed. Most households (94.5%, 518) are male-headed, with 5.5% (30 households) female-headed. Regarding educational status, 21.9% (120 household heads) have no formal education, 69.7% (382 household heads) have completed primary or secondary education, and 8.4% (46 household heads) have a college or university education. Among spouses, 30.7% (168 households) have no formal education, 64.2% (352 households) have primary or secondary education, and 5.1% (28 households) have a college or university education.

Food insecurity is prevalent, with 68.1% (373 households) experiencing insecurity, while 31.9% (175 households) are food secure. The wealth index shows that 24.5% (134 households) belong to the higher wealth category, 35.8% (196 households) to the lower category, and 39.8% (218 households) to the medium category. Land ownership is divided, with 52.2% (286 households) owning land and 47.8% (262 households) not owning land.

The mean family size is 5.1 members (SD = 1.8), with a mean age dependency ratio of 0.5 (SD = 0.2) and a mean paternal-maternal age difference of 7.6 years (SD = 6.0). The overall mean dependency ratio is 2.2 (SD = 1.3). Finally, the average land size owned by households is 0.43 hectares (SD = 22). This demographic and socioeconomic profile offers a comprehensive understanding of the community's characteristics.

**Figure 1:**
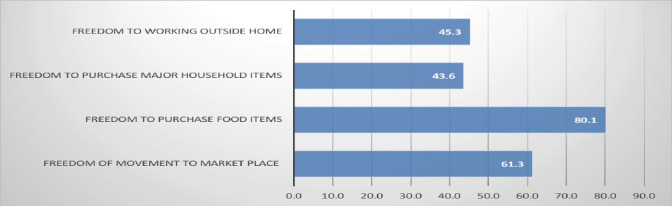
Distribution of domains of maternal financial autonomy in percent, Jimma Zone, Ethiopia

**Domains of maternal financial autonomy**: The data on maternal financial autonomy as depicted in Figure 1 shows that 61.3% (336 participants) of women have the freedom to visit marketplaces, while 38.7% (212 participants) do not. When it comes to purchasing food, 80.1% (439 participants) have the autonomy to make these purchases independently, compared to 19.9% (109 participants) who do not. However, autonomy decreases when purchasing major household items, with only 43.6% (239 participants) able to do so, while 56.4% (309 participants) cannot. Lastly, 45.3% (248 participants) of women have the freedom to work outside the home, while 54.7% (300 participants) are restricted. Of the 548 mothers interviewed, only 310 (51.6%) achieved below half of the four autonomy domains.

**Child characteristics and meal frequency**: The date in Table 2 reveals tha child sex distribution is fairly balanced, with 47.3% (259 children) being male and 52.7% (289 children) being female. In terms of meal frequency, 47.1% (258 children) meet the optimal minimum meal frequency, while 52.9% (290 children) fall into the suboptimal category.

**Table 2:** Child sex and meal frequency, Jimma Zone, Ethiopia

VARIABLE		N (%)
**CHILD SEX**	Male	259 (47.3)
	Female	289 (52.7)
**MINIMUM MEAL**	Optimal	258 (47.1)
**FREQUENCY**	Suboptimal	290 (52.9)

**Association of maternal financial autonomy and child meal frequency**: In multivariable logistic regression (Table 3), factors such as setting (OR: 0.98; 95% CI: 0.63 to 1.54), family size (Beta: 0.97; 95% CI: 0.87 to 1.08), sex of the household head (OR: 0.54; 95% CI: 0.23 to 1.24), child sex (OR: 1.34; 95% CI: 0.92 to 1.95), child age (Beta: 1.03; 95% CI: 0.99 to 1.06), marital status (OR: 1.11; 95% CI: 0.55 to 2.23), land ownership (OR: 0.83; 95% CI: 0.57 to 1.20), wealth index (lower category OR: 0.65; 95% CI: 0.40 to 1.06, middle category OR: 0.66; 95% CI: 0.42 to 1.04), dependency ratio (OR: 0.39; 95% CI: 0.10 to 1.46), household head education (no formal education OR: 1.83; 95% CI: 0.78 to 4.34, primary/secondary education OR: 1.90; 95% CI: 0.89 to 4.04), and spousal education (no formal education OR: 0.69; 95% CI: 0.25 to 1.86, primary/secondary education OR: 0.65; 95% CI: 0.26 to 1.65) showed no significant association with child minimal meal frequency. However, maternal financial autonomy had a significantly strong positive effect on a child's minimal meal frequency, with an odds ratio of 5.90 (95% CI: 3.97 to 8.78).

**Table 3:** Multivariable logistic regression on association of maternal autonomy and other socio-demographic covariates with child minimum meal frequency achievement

Variables	Exp(B)	95% C.I. for EXP(B)	

		Lower	Upper
Setting: Urban	0.98	0.63	1.54
Family size	0.97	0.87	1.08
Sex of the household head: Male	0.54	0.23	1.24
Child sex: male	1.34	0.92	1.95
Child age	1.03	0.99	1.06
Marital status: Married	1.11	0.55	2.23
Land ownership: Yes	0.83	0.57	1.20
Wealth index: higher			
Wealth index: lower	0.65	0.40	1.06
Wealth index: Middle	0.66	0.42	1.04
Dependency ratio	0.39	0.10	1.46
Household head educational status: College/University			
Household head educational status: No formal education	1.83	0.78	4.34
Household head educational status: Primary/secondary	1.90	0.89	4.04
Spousal educational status: Collage/University			
Spousal educational status: No formal educational status	0.69	0.25	1.86
Spousal educational status: Primary/secondary	0.65	0.26	1.65
Financial autonomy: above 2	5.90	3.97	8.78

## Discussion

This study on maternal financial autonomy sheds light on key aspects of maternal decision-making and child feeding practices. In the study area, many women have the ability to visit marketplaces (80%) and make independent food purchases (63%), indicating some control over daily household decisions. However, their autonomy is more limited when it comes to significant financial decisions, such as purchasing major household items, with only 43.6% of women exercising control in this area. These decisions are often made by others, particularly men in the household. Additionally, many women face significant barriers to working outside the home, further constraining their financial independence and economic opportunities. These limitations may hinder their ability to contribute to household income and provide optimal feeding for their children. The data also shows that nearly half of the children in the study area do not meet the optimal minimum meal frequency.

The multivariable logistic regression analysis reveals that factors such as setting, family size, sex of the household head, child sex, child age, marital status, land ownership, wealth index, dependency ratio, and the educational status of both the household head and spouse do not show a significant association with children's meal frequency. However, maternal financial autonomy stands out as a significantly influential factor. The strong positive effect of maternal financial autonomy on a child's minimal meal frequency suggests that greater control over financial resources enables mothers to ensure their children receive the appropriate number of meals.

While it is generally assumed that higher household income or wealth can improve a child's nutrition by providing more resources for food and essentials, global data shows that a significant proportion of stunted children come from the wealthiest households, particularly in regions like Sub-Saharan Africa and South Asia (22, 23). This raises questions about the relationship between income, wealth, and nutrition, suggesting that household income may not always directly translate into better child nutrition. In wealthier households, financial resources might not prioritize child nutrition due to factors like maternal autonomy, cultural practices, or other spending priorities (11-13). If women, who are often the primary caregivers, lack decision-making power, household wealth may be allocated in ways that do not prioritize child nutrition (12, 15). Thus, improving maternal financial autonomy is critical to ensuring that available resources are effectively used to improve child feeding habits and nutrition.

Several studies support the findings of the present study, highlighting the role of financial autonomy in influencing child meal frequency. For example, a study in Ghana found that financial autonomy, particularly the ability to make significant purchases, was an independent predictor of meeting the minimum daily meal frequency for children (24). This suggests that when women have greater control over financial resources, they are more likely to ensure their children receive adequate daily meals. Similarly, a meta-analysis of 50 Demographic and Health Surveys across low-and middle-income countries revealed that children of employed women, serving as a proxy for financial independence, had significantly higher odds of meeting the minimum meal frequency compared to children of non-employed women (25). A large-scale study across ten sub-Saharan African countries also found a positive association between women's economic empowerment and adherence to optimal child feeding practices, including meeting minimum meal frequencies (26). These studies collectively reinforce the idea that maternal economic empowerment or autonomy plays a crucial role in improving child feeding by enabling better financial decisions regarding food purchasing and meal frequency.

In such settings, promoting interventions that enhance maternal financial autonomy can help improve children's meal frequency. These interventions may include providing financial literacy and economic opportunities to women, strengthening legal frameworks to support women's control over assets, and promoting gender equality in household decision-making. Integrating financial empowerment into existing health and food security programs could offer a comprehensive approach to improving both economic and nutritional outcomes. Furthermore, community-based initiatives should engage local populations in understanding the importance of maternal financial autonomy and its impact on child nutrition, helping shift cultural norms that restrict women's economic participation. By addressing these areas, programs can be designed to more effectively enhance women's control over resources and, in turn, improve child feeding practices.

While this study focuses on maternal financial autonomy, it acknowledges that autonomy is multidimensional, as are the interventions and policies aimed at empowering women. Understanding how different dimensions of autonomy relate to feeding practices can help refine policy recommendations for more effective interventions.

In summary, the findings underscore the critical role maternal financial autonomy plays in determining children's meal frequency in the study area. This correlation suggests that women with greater control over financial resources are better positioned to ensure their children receive adequate and regular meals. Interventions in such settings should, therefore, prioritize empowering women through greater autonomy over resources, alongside efforts to improve health and food security. This dual approach can create a more supportive environment for families and ultimately lead to improved nutritional outcomes for children.

The current study employed a cross-sectional design, which is useful for examining associations among variables. However, such studies are limited in their ability to establish causality. As the data provides only a snapshot of feeding patterns for different children at a single point in time, it is difficult to capture changes in variables over time. Future studies would benefit from longitudinal data and experimental designs to more robustly establish causal relationships between maternal autonomy measures and feeding outcomes.
